# Supplementary material for: Identification of photoperiod-induced specific miRNAs in the adrenal glands of Sunite sheep (Ovis aries)
Source: Front Vet Sci. 2022 Jul 22;9:888207. doi: 10.3389/fvets.2022.888207 (PMC9354845; doi:10.3389/fvets.2022.888207)
Supplement: Supplementary file 1 [file Data_Sheet_1.ZIP › Supplementary file/miRDeep2 file.zip!/miRDeep2 file/Know_Structure/SP42A3/oar-mir-221.pdf]

miRBase precursor : oar-mir-221
Total read count : 1775
oar-miR-221 read count : 1540
remaining reads : 235

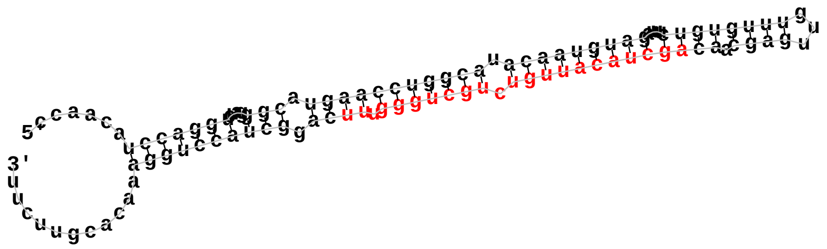

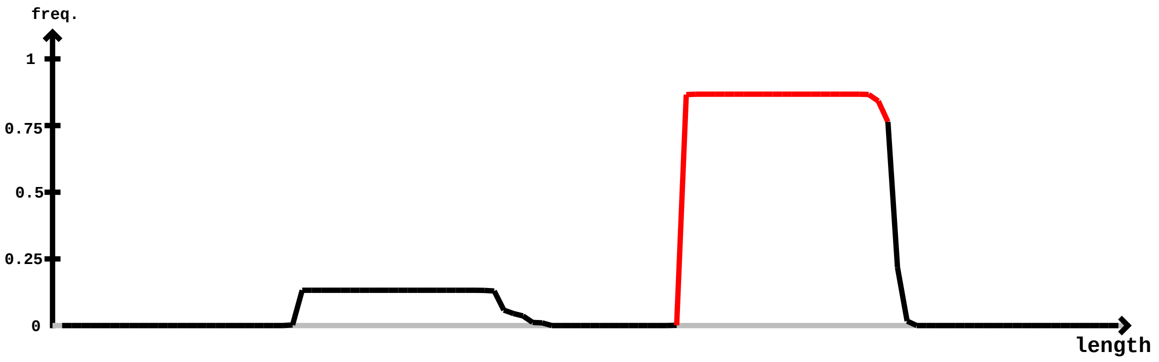

oar-miR-221

```
5'- ccaacauccaggucuagggcaugaaccuggcauacaauguagauuucuguguuuguugagcaacagcuacauugucugcuggguuucaggcuaccuggaaacacguucuu   -3'   exp
    ......(((((((....(((.(((((((((((.(((((((((....((((((((...)))).))))))))))))).))))))).)))).))))))))))...........   reads   mm   sample
    .......................aaccuggcauacaauguagauu.................................................................   1       0    seq
    .......................aaccuggcauacaauguagauuu................................................................   1       0    seq
    .......................aaccuggcauacaauguagauuuc...............................................................   1       0    seq
    .......................aaccuggcauacaauguagauuucugu............................................................   1       0    seq
    ........................accuggcauacaauguaga...................................................................   1       0    seq
    ........................accuggcauacaauguagau..................................................................   4       0    seq
    ........................accuggcauacaauguagauu.................................................................   126     0    seq
    ........................accuggcauacaauguagauuu................................................................   22      0    seq
    ........................accuggcauacaauguagauuuc...............................................................   15      0    seq
    ........................accuggcauacaauguagauuucu..............................................................   43      0    seq
    ........................accuggcauacaauguagauuucug.............................................................   3       0    seq
    ........................accuggcauacaauguagauuucugu............................................................   17      0    seq
    ...............................................................cagcuacauugucugcuggguuu........................   1       0    seq
    ...............................................................cagcuacauugucugcuggguuuc.......................   1       0    seq
    ................................................................agcuacauugucugcuggg...........................   2       0    seq
    ................................................................agcuacauugucugcugggu..........................   44      0    seq
    ................................................................agcuacauugucugcuggguu.........................   137     0    seq
    ................................................................agcuacauugucugcuggguuu........................   967     0    seq
    ................................................................agcuacauugucugcuggguuuc.......................   355     0    seq
    ................................................................agcuacauugucugcuggguuuca......................   30      0    seq
    .................................................................gcuacauugucugcuggguu.........................   1       0    seq
    .................................................................gcuacauugucugcuggguuu........................   2       0    seq
```
